# How Does Glycation Affect Binding Parameters of the Albumin-Gliclazide System in the Presence of Drugs Commonly Used in Diabetes? In Vitro Spectroscopic Study

**DOI:** 10.3390/molecules26133869

**Published:** 2021-06-24

**Authors:** Katarzyna Wiglusz, Ewa Żurawska-Płaksej, Anna Rorbach-Dolata, Agnieszka Piwowar

**Affiliations:** 1 Department of Analytical Chemistry, Wroclaw Medical University, Borowska 211, PL-50556 Wrocław, Poland; katarzyna.wiglusz@umed.wroc.pl; 2 Department of Toxicology, Wroclaw Medical University, Borowska 211, PL-50556 Wrocław, Poland; anna.rorbach-dolata@umed.wroc.pl (A.R.-D.); agnieszka.piwowar@umed.wroc.pl (A.P.); 3 Department of Pharmaceutical Biochemistry, Wroclaw Medical University, Borowska 211, PL-50556 Wrocław, Poland

**Keywords:** albumin-drug interactions, bovine serum albumin, gliclazide, acetylsalicylic acid, atorvastatin, cilazapril, glycation, binding affinity

## Abstract

In this research, the selected drugs commonly used in diabetes and its comorbidities (gliclazide, cilazapril, atorvastatin, and acetylsalicylic acid) were studied for their interactions with bovine serum albumin—native and glycated. Two different spectroscopic methods, fluorescence quenching and circular dichroism, were utilized to elucidate the binding interactions of the investigational drugs. The glycation process was induced in BSA by glucose and was confirmed by the presence of advanced glycosylation end products (AGEs). The interaction between albumin and gliclazide, with the presence of another drug, was confirmed by calculation of association constants (0.11–1.07 × 10^4^ M^−1^). The nature of changes in the secondary structure of a protein depends on the drug used and the degree of glycation. Therefore, these interactions may have an influence on pharmacokinetic parameters.

## 1. Introduction

Diabetes is recognized as the major lifestyle disease in the modern world, currently affecting over 450 million people. Higher blood glucose level is the most known symptom. Diabetes is also a risk factor for cardiovascular diseases. A diagnosis of diabetes implies the need to implement multidrug therapy—not only hypoglycemic but also hypotensive, lipid-lowering, and antiplatelet therapy [1,2]. It generates the possibility of drug-to-drug interactions and may cause unpredictable effects in their pharmacodynamic and pharmacokinetics [3]. In the context of diabetes, hyperglycemia leads to exacerbated non-enzymatic attachment of the glucose molecule to plasma proteins in a process called glycation, which affects their structure, biological function, and their binding properties. It has been reported that glycated albumin has a decreased affinity for fatty acids, which may have serious biochemical consequences, e.g., platelet hyperactivity [4]. Diabetic patients are considered to have a 2–3-fold higher level of glycated albumin compared with healthy subjects [5]. Since many pharmacological agents extensively bind to plasma albumin, it is important to know how this process can be altered under hyperglycemic conditions and in the presence of other drugs (protein-displacing interactions), and such investigations are intensively conducted [6,7,8,9].

**Figure 1:**
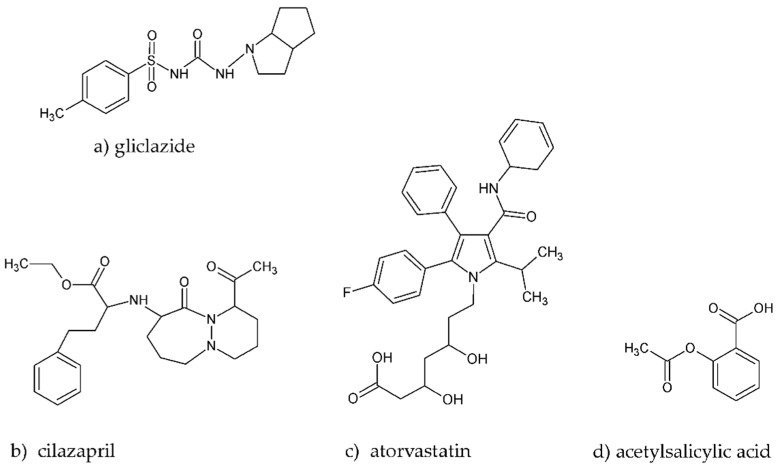
Chemical structures of gliclazide (**a**), cilazapril (**b**), atorvastatin (**c**) and acetylsalicylic acid (**d**).

Second-generation sulphonylureas are still widely used for hypoglycemic treatment [10]. Our previous study revealed that gliclazide (GLICL, Figure 1a), which belongs to this group, binds more weakly to glycated bovine serum albumin (BSA) than to its native form in the BSA-GLICL binary system, which may cause higher free-drug concentration in systemic circulation [11]. Here we focused on the spectroscopic evaluation of the conformational behavior of differently glycated albumin upon binding to gliclazide (as a ‘basal’ agent) in the co-presence of another drug in ternary systems (BSA-DRUG-GLICL). The aim was realized by fluorescence quenching and circular dichroism. These techniques are generally accepted for studying in vitro interactions of ligands with plasma albumin because of their many advantages: sensitivity, rapidity, and simplicity [12,13,14]. They are very useful for the calculation of binding parameters and mean residue ellipticity [15,16]. A previously developed model of glycated BSA, simulating a non-diabetic state (g10_BSA, albumin exposed to 10 mM glucose) and poorly controlled diabetes (g30_BSA, albumin exposed to 30 mM glucose) was applied in the present study [11]. From a variety of oral drugs used in the treatment of diabetes comorbidities, we chose three commonly applied agents for investigation in this study: cilazapril (CIL), an angiotensin-converting enzyme inhibitor with hypotensive activity (Figure 1b); atorvastatin (ATOR), a 3-hydroxy-methylglutarl coenzyme A reductase inhibitor, used to lower serum cholesterol level (Figure 1c); acetylsalicylic acid (ASA), an antiplatelet agent that inhibits cyclooxygenase-1 (Figure 1d) [17,18,19,20]. To our knowledge there is no data on such drug combinations in the scientific literature.

## 2. Results and Discussion

### 2.1. BSA Glycation

**Figure 2:**
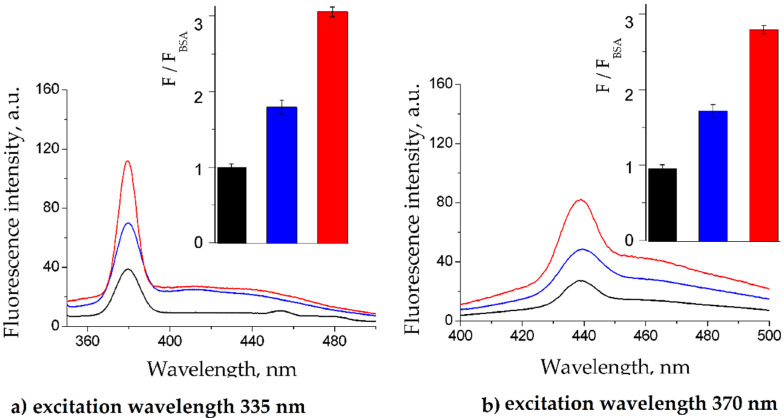
Emission spectra of nonglycated and differently glycated BSA at excitation wavelength 335 nm (**a**) and 370 nm (**b**). Relative increase in fluorescence intensity is shown in the inserts. Native BSA (black lines and bars), g10_BSA (blue lines and bars) and g30_BSA (red lines and bars) signify the use of 0, 10 and 30 mM glucose for glycation.

Reducing sugars react non-enzymatically with amino groups of protein and, as a result of different rearrangements, cross-linked, heterogeneous fluorescent derivatives are formed. In vitro studies employ several glycation agents and various experimental conditions to initiate protein glycation. Although glucose reveals rather weak glycative properties, its use was justified by the fact that it is the most prevalent endogenous reducing sugar [6,21]. Figure 2 shows emission spectra collected at 335 and 370 nm excitation wavelength. An observed increase in fluorescence intensity (FI) at 380 (Figure 2a) and 440 nm (Figure 2b), dependent on glucose concentration, is characteristic for glycation products containing lysine-arginine- or lysine-derived cross-links [22]. Hereby, we confirmed the effectiveness of glycation under the experimental conditions applied in this study.

### 2.2. Fluorescence Quenching of BSA by Gliclazide in the Presence of Cilazapril, Atorvastatin, and Acetylsalicylic Acid

**Figure 3:**
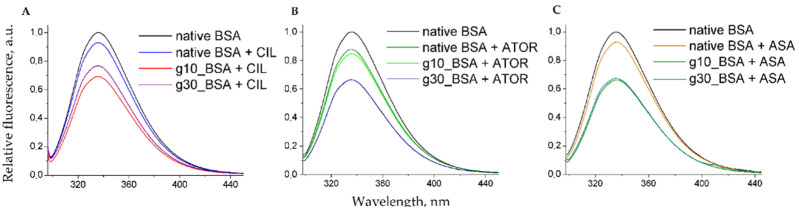
Fluorescence emission spectra of native BSA, g10_BSA and g30_BSA in the presence of cilazapril (**A**), atorvastatin (**B**) and acetylsalicylic acid (**C**) recorded at λ_ex_ 295 nm.

Fluorescence spectroscopy, although known for a long time, is still applied in the newest research for studying ligand-protein interactions, and we too used this valuable tool [15,23]. Firstly, the fluorescence spectra of native BSA, g10_BSA, and g30_BSA were recorded in the presence of cilazapril, atorvastatin, and acetylsalicylic acid (which are further referred to as DRUG). Results are presented in Figure 3. Intrinsic fluorescence derived from tryptophan residues decreased after the addition of ligands, which indicates formation of protein-drug complexes. It can be clearly observed that intensity of fluorescence decreased with the glycation of BSA: native BSA-DRUG > g10_BSA-DRUG > g30_BSA-DRUG, indicating changes in the protein’s environment induced by glucose attachment. This trend is similar to interactions of gliclazide with glycated albumin, as shown in a previous study, and remains in accordance with results obtained by other researchers, as well as for human serum albumin (HSA) [11,24,25].

**Figure 4:**
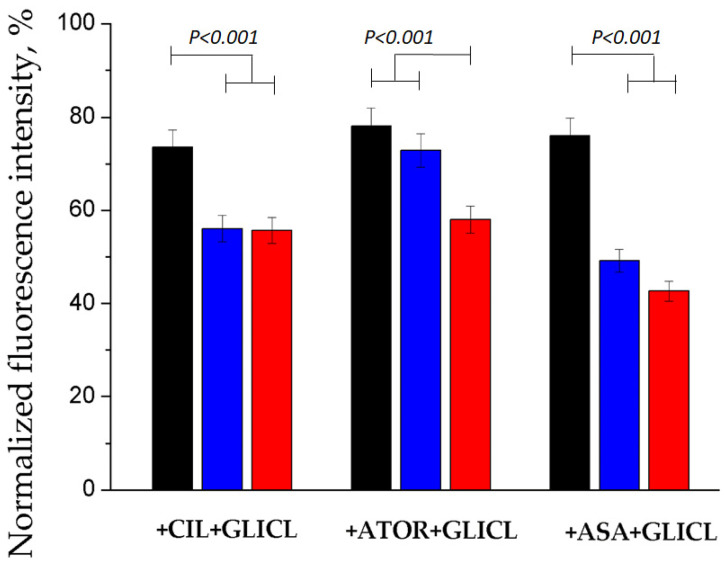
Changes in fluorescence intensity of BSA solutions (nonglycated and glycated at different degrees) after the addition of 14 μM gliclazide (molar ratio 1:7) in the presence of another drug observed at 295 nm excitation wavelength. Fluorescence is normalized to the value observed for the BSA-DRUG (cilazapril, atorvastatin or acetylsalicylic acid) complex and expressed as percentage. The colors of the bars refer to the following: native BSA—black, g10_BSA—blue, g30_BSA—red. Statistical significance was calculated using ANOVA, and *P* < 0.05 was considered significant.

In the next step, we focused on fluorescence quenching of BSA (non-glycated and glycated) caused by gliclazide in the presence of examined drugs (ternary complex model). This approach enables the investigation of both the effect of glycation and possible binding interactions of GLICL. Gliclazide is highly bound to serum albumin (up to 97%) and has a relatively low volume of distribution. Thus, even small changes in the structure of albumin may cause substantial changes in its pharmacokinetics. Amongst different hypoglycemic drugs, gliclazide shows the most significant decrease in binding to albumin when bundled with competing drugs [26,27]. Detailed fluorescence quenching spectra of native BSA, g10_BSA and g30_BSA (with fixed protein concentration) were recorded upon the increase in concentration of GLICL in the presence of a second compound and are available in Supplementary Materials (Figure S1). The obtained data demonstrated a gradual decrease in FI derived from fluorophores of the BSA molecule (tyrosyl and tryptophanyl residues) upon increasing GLICL concentration, indicating the formation of ternary complexes [28]. Moreover, the decrease in FI deepened with the degree of glycation, being the most pronounced for g30_BSA, which mimics diabetic conditions. We did not observe any shifts in emission maxima, which were around 335–337 nm. Spectra curves were characterized by a similar shape and trend of changes for both excitation wavelengths (280 and 295 nm). Therefore, we conclude that the contribution of tyrosyl residues in the interaction of DRUG-GLICL is negligible, independently from glycation. Szkudlarek et al. [9] have shared a similar observation with regard to gliclazide and glycated HSA. Since BSA has two tryptophanyl residues (Trp134 and Trp213), this phenomenon is even more pronounced than for HSA with a single tryptophan. However, Trp134 is considered less important because of its position on the subdomain IB surface, whereas Trp213 is located in the subdomain IIA hydrophobic pocket [29,30]. Taking under account the fluorescent behavior of BSA, only results for an excitation wavelength of 295 nm are presented in the following parts of this work. Figure 4 summarizes the reducing effect of the GLICL on fluorescence intensity. Interestingly, the difference in FI between g10_BSA and g30_BSA is not very big (and without statistical significance), at least for cilazapril and acetylsalicylic acid, so it can be concluded that these interactions would have rather poor clinical relevance. As for atorvastatin, the difference of FI is significant, and could indicate potential ATOR-GLICL interaction in diabetic patients. However, such an effect is not evidenced in clinical practice.

**Figure 5:**
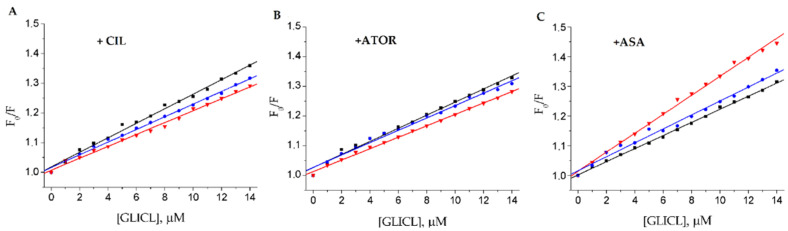
The Stern-Volmer plots for the interaction of gliclazide with BSA in the presence of ci-lazapril (**A**), atorvastatin (**B**) and acetylsalicylic acid (**C**): native BSA (■), g10_BSA (●), g30_BSA (▲) at λ_ex_ = 295 nm.

Obtained fluorescence data was used to calculate binding parameters of the PROTEIN-DRUG-GLICL system. Stern-Volmer quenching constants (K_SV_) were determined by the intercept of the linear regression plot graph of the relative emission intensity, as shown in Figure 5. The plots showed that the results exhibited a good linear relationship within the investigated concentration. The effect of the quencher, on the intensity of albumin fluorescence, is clearly observed, and the kq value is greater than the diffusion rate constant of the biopolymer (2.0 × 10^10^ L/mol s), which also proves the formation of ternary complexes [31]. Directional coefficients, characterizing curves of glycated BSA, are smaller than for native protein. Based on log transformations of those graphs (provided in Supplementary Materials as Figure S2), binding constants (K_a_), and the number of bound gliclazide molecules (n) were calculated, and results are presented in Table 1.

K_SV_ values reveal different changes depending on the drug model. For GLICL in the presence of CIL, they decrease gradually along with the glycation degree, but in the presence of ATOR they show the opposite trend. The growth of the *K_SV_* value is associated with the increase in ligand molecule availability to the macromolecule and the formation of the complex in an excited state [32]. One possible explanation is that glycated protein has an altered structure, and it may somehow facilitate the formation of a PROTEIN-GLICL complex in the presence of atorvastatin. However, obtained differences in *K_SV_* values between nonglycated and glycated BSA are not big—typically 10–20%—and for ASA-GLICL they reach 30% for g10_BSA, which is difficult to explain. Comparing K_SV_ in g10_BSA and g30_BSA models (reflecting physiological and diabetic conditions) the differences are even smaller (4–6%). It should be also underlined that all values of K_SV_ obtained in those ternary models are lower than values for the PROTEIN-GLICL system without the presence of a second drug (7.81 × 10^4^, 6.83 × 10^4^ and 3.25 × 10^4^ M^−1^ for native, g10 and g30_BSA, respectively), which, again, confirms a decrease in the availability of gliclazide to bind with protein [11]. When analyzing values of binding constants, they varied for native BSA and glycated models, which emphatically proves that glycation of BSA affected the affinity of the protein for the GLICL-DRUG complex. The average binding constant values usually lie in the range of 1–15 × 10^4^ M^−1^. In our study K_a_ for ternary complexes they varied from 0.11 to 1.07 × 10^4^ M^−1^. Similar effects and variations, in affinity at comparable levels of glycation, have been noted in recent studies examining the binding of sulfonylureas with glycated HSA both in vitro and in vivo [9,33]. On the other hand, some researchers observed increased K_a_ values upon glycation [25,34]. Such results were obtained for PROTEIN-ATOR-GLICL. It may have happened that the affinity of GLICL to different binding sites changed, due to glycation, as has been documented for another sulfonylurea drug [35]. We did not investigate the exact competitive effect of gliclazide and specific site markers in glycated BSA, but given the high protein binding of atorvastatin (up to 98%) and its preferences for subdomain IIA, it is possible that a displacement of GLICL from Sudlow site I to site II occurred [36]. Therefore, it can be stated with confidence that glycation of BSA affected the affinity of the protein to form a complex, but it may lead to either an increase or decrease in affinity at a given site [37]. It should also be mentioned that, in comparison to our previous experiment on the effect of albumin glycation on gliclazide binding, the strength of binding of GLICL in the presence of an additional compound is at least 3-fold lower [11]. In this light, albumin glycation did not cause as large of changes in binding as the presence of other drugs. This is consistent with the general knowledge that the probability of drug interactions increases with the growing number of drugs per person, and it also provides evidence for the clinical relevance of protein-drug-drug interaction, as well as an explanation for the rationale of binding studies as the first step for prediction of pharmacokinetic properties and adverse drug effects (hypoglycemic episodes in this particular study). We have previously reported K_a_ of GLICL and BSA, g10_BSA, and g30_BSA complexes as 3.80, 2.52, and 1.15 × 10^4^ M^−1^, respectively [11]. Interestingly, in ternary complexes investigated in this study, the impact of additional drugs (cilazapril, atorvastatin, or acetylsalicylic acid) on the binding of gliclazide with serum albumin was the most evident in the native model (4, 8, and 35-fold decrease for ASA, CIL, and ATOR, respectively, in comparison with the binary model), while it was only slightly marked in the diabetic model (3, 5, and 5-fold decrease for ASA, CIL, and ATOR, respectively, in comparison with the binary model), which is compatible with the observations of Szkudlarek et al. [34]. Since under physiological conditions serum albumin is also exposed to glucose, we consider it more appropriate to compare g10_BSA with the g30_BSA model. From that point of view, the most significant changes in binding of gliclazide were observed in the presence of ASA. In fact, numerous in vivo studies confirmed that the hypoglycemic effect of gliclazide and other sulfonylureas is enhanced by concomitant administration of acetylsalicylic acid [37,38,39,40]. As indicated in Table 1, the *n* values are in a range of 0.7–0.9, suggesting that GLICL binds to BSA with moderate affinity at about 1:1 stoichiometry, independently of the presence of other drugs tested. This may confirm our assumptions that GLICL is displaced from the high-affinity binding site, but it is still bound to albumin [36]. Based on the presented results regarding the binding interactions, of drugs commonly used in diabetes, to glycated albumin, it can be assumed that glycation may modify drug response and affect their pharmacokinetics. This knowledge is particularly important in polypharmacy, when risk of incidence of drug-drug interactions is much higher. Literature data shows that diabetic patients take, on average, even different drugs, which requires individualization of therapeutic strategies [41]. Decreased binding of drugs towards glycated albumin may increase their pharmacological activity and predispose to occurrence of life-threatening side effects, especially in elderly patients [42].

### 2.3. Changes to α-Helical Contents

**Figure 6:**
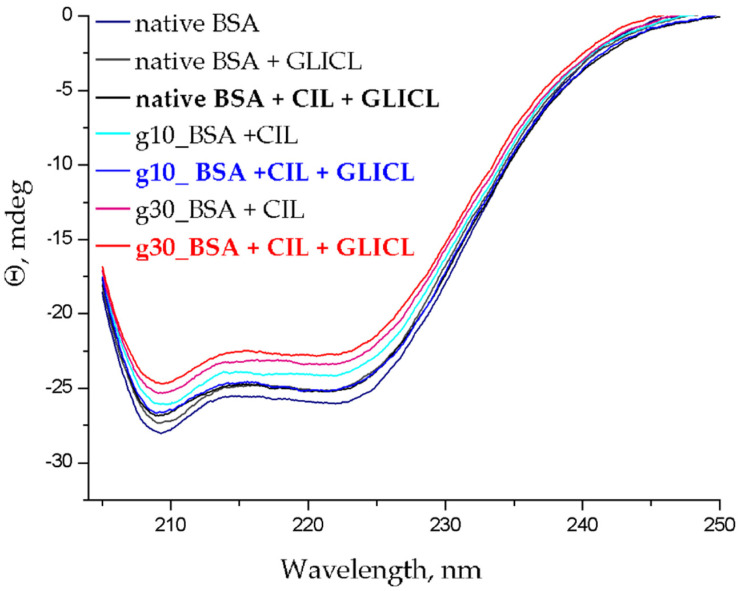
Circular dichroism spectra of protein (2 μM): native BSA, g10_BSA, g30_BSA with CIL (8 μM) and GLICL (14 μM), pH 7.4.

Alterations in the far UV CD spectrum reflect changes of the proteins’ secondary structure triggered by ligands binding [43]. The CD spectrum of BSA exhibits two negative bands, characteristic for α-helix structure, at 208 and 222 nm. Detailed CD spectra of nonglycated and differentially glycated BSA, incubated with gliclazide in the presence of competitive drugs, are provided in Supplementary Materials Figure S3. Figure 6 shows a representative example of observed changes. Summarized results of percentage changes of α-helical contents, calculated based on the equation in Section 3.4, are presented in Table 2.

The shape of peaks, and the peak maximum position, remained almost the same, which may indicate that BSA has a predominantly α-helix nature even after binding to the drug. The content of secondary structures (α-helix) of BSA after glycation was increased from 2.14 to 3.84 and from 1.88 to 3.61 for the interaction with DRUG (CIL and ATOR) and GLICL, respectively. However, the α-helical structure for protein with ASA and GLICL decreased from 4.79 to 2.05. A similar effect was noticed in glycated hemoglobin [44]. These results suggest that ATOR and CIL unfolded albumin (native and glycated protein), and ASA have a slight stabilizing effect on the secondary structure for glycated albumin. An analogous observation was described by Quiming et al. [45] when studying the interaction between BSA and metallothionein (increase in α-helix upon addition of the ligand). This indicates that the glycation process may cause a different effect on the secondary structure of the protein.

## 3. Materials and Methods

### 3.1. BSA Glycation

Bovine serum albumin (≥96%) was used to prepare 1.2 mM stock solution in PBS (pH 7.4), preserved with 0.1% sodium azide. Native and differently glycated albumin solutions were then prepared by treatment with PBS (non-glycated BSA), 10 mM glucose (g10_BSA) and 30 mM glucose (g30_BSA), followed by incubation at 37 °C for 21 days [11]. The products of glycation were analyzed through the fluorescence experiment (excitation wavelength 335 and 370 nm) using the Hitachi F-7000 spectrophotometer (Tokyo, Japan). All measurements were performed in triplicate.

### 3.2. Binding Experiments

**Scheme 1:**
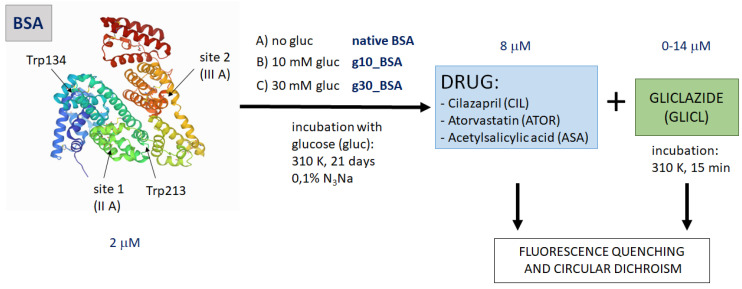
Schematic work for the study of binding interactions of the albumin-gliclazide-drug system [46].

One mM stock solution of gliclazide and 0.4 mM stock solutions of cilazapril, atorvastatin, and acetylsalicylic acid were prepared in 5% methanol/PBS. Albumin solutions: BSA, g10_BSA, and g30_BSA were pre-incubated with CIL, ATOR, or ASA solution (final molar ratio DRUG to albumin 4:1), then GLICL was added to the mixtures in increasing concentrations (final molar ratio GLICL to albumin from 0:1 to 7:1) and incubated for 15 min in 310 K. The schematic workflow is presented in Scheme 1. Quenching experiments and CD measurements were repeated in triplicate.

All reagents were received from Sigma-Aldrich (St. Luis, MO, USA).

### 3.3. Fluorescence Quenching and Calculation of Binding Parameters

To analyze the changes in the tertiary structure of all BSA solutions, after binding with GLICL and other ligands (CIL, ATOR, or ASA), fluorescence studies were conducted (Hitachi F-7000 spectrophotometer) at excitation wavelengths of 280 and 295 nm with the slit widths set at 5 nm A quartz cuvette with a path length of 1 cm was used. The scanning speed was 200 nm/min. Each spectrum background was corrected by subtracting a spectrum of the phosphate buffer as a blank sample. Fluorescence spectra of drugs alone were also recorded.

The fluorescence intensity was corrected for energy reabsorption (internal filter effect). Measurements of absorbance at the excitation and emission wavelength used were made with a Perkin Elmer Lambda 20 spectrophotometer (Waltham, MA, USA). The corrected fluorescence intensity (F_cor_) values were obtained by the following Equation (1) [15]:

(1) $$F_{cor}=F_{obs}\cdot 10^{\left(A_{ex}+A_{em}\right)/2}$$

where *F_cor_* and Fobs are corrected and observed fluorescence, respectively; *A_ex_* and *A_em_* are the absorbance at the excitation and emission wavelength, respectively.

The magnitude of fluorescent quenching of the protein, induced by tested compounds, was calculated according to the Stern-Volmer Equation (2):

(2) $$\frac{F_{0}}{F}=1+k_{q}\tau_{0}=1+K_{\mathrm{SV}}Q$$

where *F* and *F*_0_ are the fluorescence intensity with and without the quencher (GLICL), respectively; *k_q_* is the quenching rate constant; *τ*_0_ is the average lifetime of a biomolecule without the quencher (*τ*_0_ = 10^−8^ s); *K*_SV_ is the Stern-Volmer quenching constant (*k_q_* = *K*_SV_/*τ*_0_); *Q* is the concentration of the quencher [47,48,49].

Binding parameters (binding constant and number of binding sites) were obtained from double log modified Stern-Volmer Equation (3):

(3) $$\log\frac{F_{0}-F}{F}=\log K_{a}+n\log Q$$

where *F*_0_ and *F* are fluorescence intensities in the absence and presence of gliclazide, respectively; *K_a_* is the binding constant of the complex BSA-GLICL-DRUG, expressed as M^−1^; *n* is number of bound gliclazide; *Q* is the concentration of the quencher (GLICL).

### 3.4. α-Helical Content of Albumin Molecules

To analyze the changes in the secondary structure of all BSA solutions after binding with GLICL-DRUG, circular dichroism spectra data were determined (JASCO J-1500 spectropolarimeter (Tokyo, Japan). Measurements were recorded in a range of 205–250 nm, with a scanning speed of 200 nm/min and cell length path of 0.1 cm. This provides information about only α-helical structure. The PBS buffer absorbs energy strongly and has a significant influence on the signal-to-noise ratio (S/N), and the measurements below 195 nm cannot be performed for complete secondary structure analysis in such experimental conditions. The baseline was corrected using a phosphate buffer, pH = 7.4. Mean residue ellipticity (MRE) was calculated according to the following Equation (4) and expressed in deg cm^2^ dmol^−1^:

(4) $$\mathrm{MRE}=\frac{\mathrm{observedCD}\left(m\deg\right)}{c_{p}\cdot n\cdot l\cdot 10}$$

where *C_p_* is the molar concentration of BSA, *n* is the number of amino acid residues (583 for BSA), and *l* is the path-length (0.1 cm).

The α-helical contents of free and modified BSA were calculated from MRE values at 208 nm with Equation (5):

(5) $$\alpha -\mathrm{helix}\left(\%\right)=\frac{{\mathrm{MRE}}_{208}-4000}{33000-4000}\times 100$$

where MRE_208_ is the observed MRE value at 208 nm, 4000 is the MRE of the β-form and random coil conformation cross at 208 nm, and 33,000 is the MRE value of the pure α-helix of BSA at 208 nm.

### 3.5. Statistical Analysis

Data is presented as mean ± standard deviation and statistical significance of differences between systems (native BSA, gly10_BSA, and gly30_BSA) was calculated with ANOVA and the Tukey post-hoc test.

## 4. Conclusions

The interactions of cilazapril, atorvastatin, or acetylsalicylic acid and the BSA molecule (both native and glycated) were confirmed by fluorescence studies and circular dichroism spectroscopy studies. Analysis of the circular dichroism spectra and fluorescence spectra revealed structural changes and microenvironmental perturbation around protein fluorophores upon adding ATOR, CIL, and ASA to complexes of PROTEIN-GLICL. The affinity of the albumin towards gliclazide strongly depended on the presence of the studied competing drugs. Glycation of BSA moderately affected the affinity of the protein for DRUG-GLICL complex. As observed in ternary complexes, the impact of adding the drug on the binding of gliclazide with serum albumin was the most evident in the native model. The affinity of GLICL to albumin, in the presence of ATOR, depends on the level of glycation and increases from native BSA to the glycated form (g10_BSA and g30_BSA). It was probably due to glycation and the formation of AGEs products, which could displace gliclazide or reduce its interaction with the protein. The combination of gliclazide, with the examined competitive drugs (CIL and ASA), results in a decrease in its binding constant and may increase the risk of hypoglycemia in diabetic patients, which should be taken under consideration in polypharmacy.

## Figures and Tables

**Table 1:** The calculated values of Stern-Volmer quenching constant (K_SV_), binding constant (K_a_) and number of bound gliclazide (n) for tested ternary drug models at λ_ex_ 295 nm (mean ± SD).

Drug Model	Sample Type	K_SV_ 10^4^ [M^−1^]	r	K_a_ 10^4^ [M^−1^]	n	r
CIL-GLICL	native BSA	2.45 ± 0.25	0.9929	0.48 ± 0.05	0.85 ± 0.03	0.9933
	g10_BSA	2.14 ± 0.18	0.9961	0.25 ± 0.02	0.81 ± 0.02	0.9975
	g30_BSA	2.01 ± 0.14	0.9948	0.23 ± 0.04	0.81 ± 0.04	0.9777
ATOR-GLICL	native BSA	1.93 ± 0.28	0.9904	0.11 ± 0.02	0.82 ± 0.03	0.9904
	g10_BSA	2.09 ± 0.10	0.9918	0.15 ± 0.01	0.76 ± 0.02	0.9972
	g30_BSA	2.21 ± 0.08	0.9976	0.25 ± 0.02	0.73 ± 0.02	0.9966
ASA-GLICL	native BSA	3.20 ± 0.31	0.9958	1.07 ± 0.09	0.90 ± 0.05	0.9983
	g10_BSA	2.21 ± 0.22	0.9984	0.88 ± 0.07	0.92 ± 0.04	0.9946
	g30_BSA	2.35 ± 0.44	0.9897	0.37 ± 0.05	0.84 ± 0.04	0.9835

**Table 2:** The percentage of changes (mean ± SD) to the α-helical structure of native and glycated BSA-GLICL complexes before and after interaction with DRUG (CIL, ATOR, ASA).

Drug Model	Sample Type	α-Helical Content Decrease (%)
CIL-GLICL	BSA	2.14 ± 0.11
	g10_BSA	3.84 ± 0.18
	g30_BSA	3.84 ± 0.20
ATOR-GLICL	BSA	1.88 ± 0.08
	g10_BSA	2.45 ± 0.14
	g30_BSA	3.61 ± 0.14
ASA-GLICL	BSA	4.79 ± 0.21
	g10_BSA	2.77 ± 0.19
	g30_BSA	2.05 ± 0.09

## Data Availability

Data supporting reported results are available from the corresponding author upon reasonable request.

## Supplementary Materials

The following are available online, Figure S1: Fluorescence quenching spectra of BSA, g10_BSA and g30_BSA; Figure S2: The double-logarithm plot for the interaction of gliclazide with BSA in the presence of second drug: cilazapril, atorvastatin, acetylsalicylic acid; Figure S3: Far CD-spectra of native BSA, g10_BSA and g30_BSA incubated with gliclazide in presence of cilazapril, atorvastatin, acetylsalicylic acid.

## Sample Availability

Samples of the compounds are not available from the authors.
