# Supplementary material for: How Does Glycation Affect Binding Parameters of the Albumin-Gliclazide System in the Presence of Drugs Commonly Used in Diabetes? In Vitro Spectroscopic Study
Source: Molecules. 2021 Jun 24;26(13):3869. doi: 10.3390/molecules26133869 (PMC8270297; doi:10.3390/molecules26133869)
Supplement: Supplementary file 1 [file molecules-26-03869-s001.zip › molecules-1249851-supplementary.pdf]

Supplementary Materials

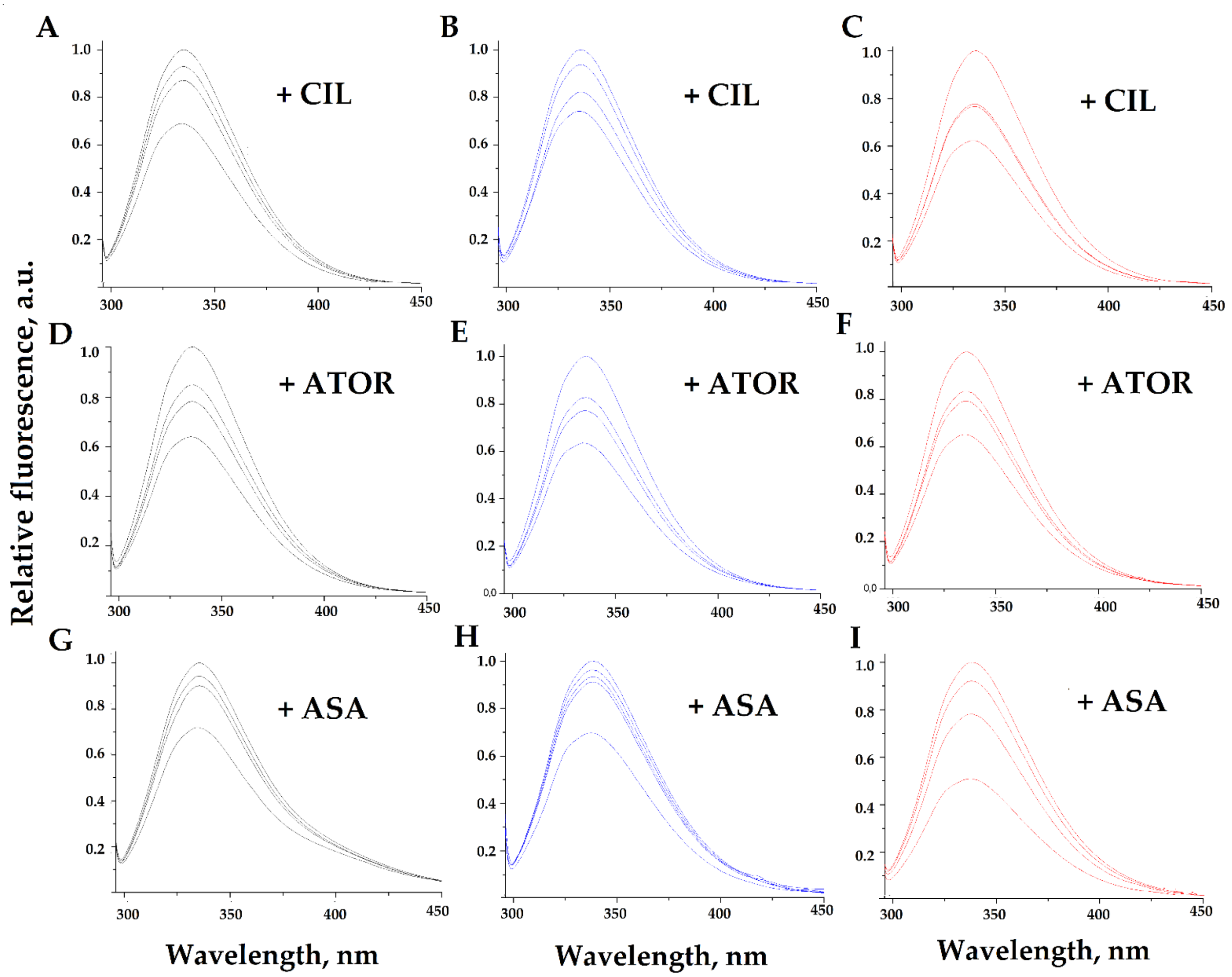

Figure S1. Fluorescence quenching spectra of BSA (**A**,**D**,**G**), g10_BSA (**B**,**E**,**H**) and g30_BSA (**C**,**F**,**I**) shown in order from the top: protein without any ligand (2 µM), protein with DRUG (cilazapril, atorvastatin, acetylsalicylic acid) in concentration of 8 µM (binary complexes, PROTEIN-DRUG), and protein and gliclazide (in the lowest and the highest concentration tested, 2 and 14 µM) in the presence of second drug (ternary complexes, PROTEIN-DRUG-GLICL).

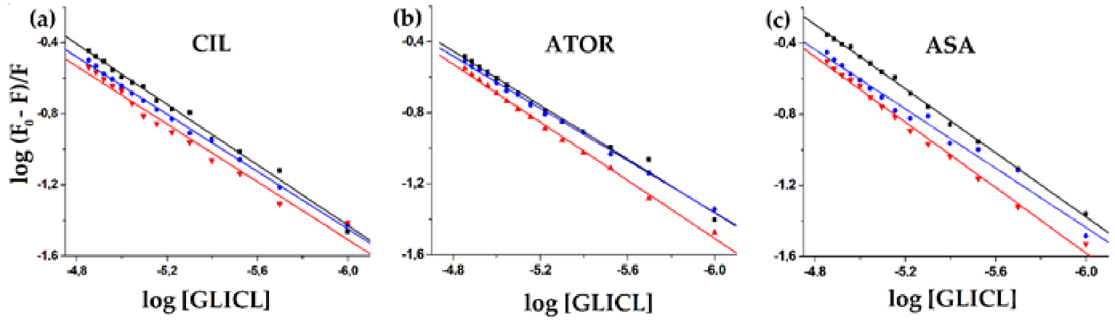

Figure S2. The double-logarithm plot for the interaction of gliclazide with BSA in the presence of second drug: cilazapril (**a**), atorvastatin (**b**), acetylsalicylic acid (**c**), λ_{ex}=295 nm. Native BSA (■), g10_BSA (●), g30_BSA (▲).

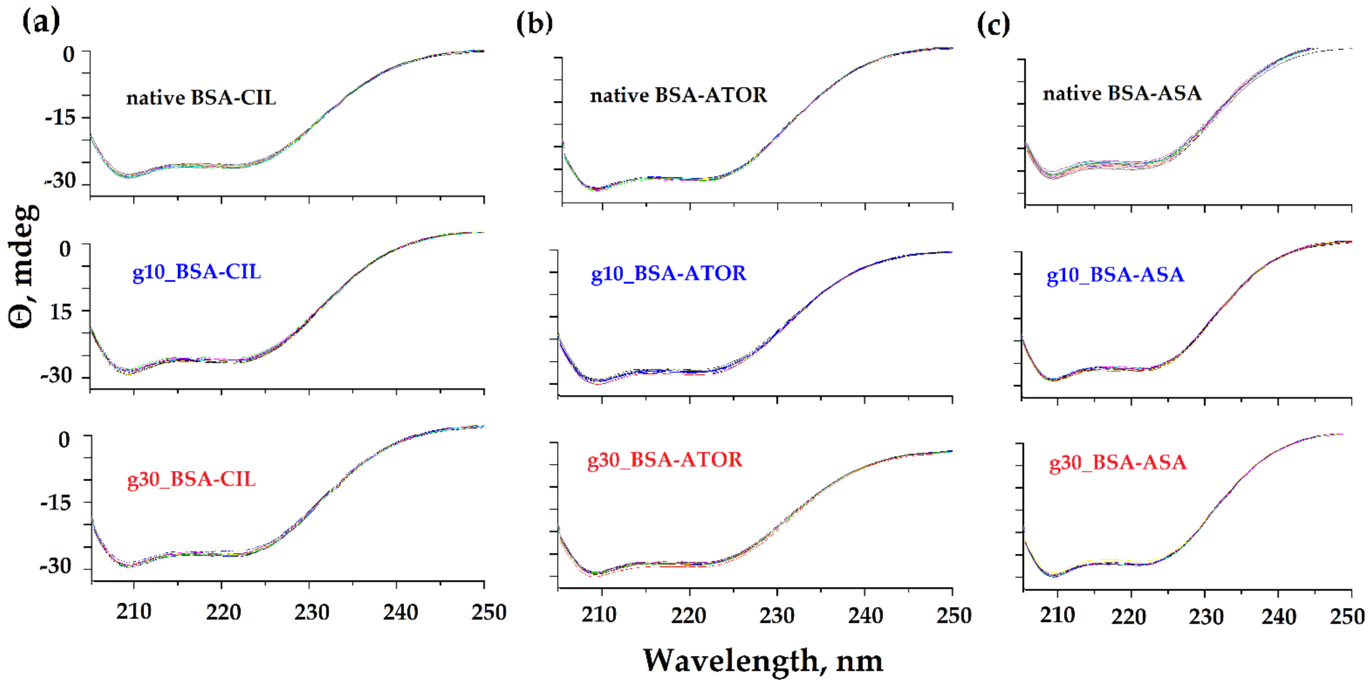

Figure S3. Far CD-spectra of native BSA, g10_BSA and g30_BSA incubated with gliclazide in presence of cilazapril (**a**), atorvastatin (**b**), acetylsalicylic acid (**c**).
